# Designing and evaluating large language model-enabled clinical decision support for heart failure: a modular and risk-tiered framework

**DOI:** 10.3389/fdgth.2026.1730457

**Published:** 2026-06-04

**Authors:** Wenfang Zhu, Jin Peng, Zhi Yan, Yuhong Chen, Jinpeng Xu, Liang Zhang

**Affiliations:** 1 Department of Cardiology, Anhui Chest Hospital, Anhui, China; 2 West China College of Basic Medicine and Forensic Medicine, Sichuan University, Sichuan, China; 3 Department of Transformation of Scientific and Technological Achievements, Capital Medical University, Beijing, China; 4 School of Medical Technology, Sichuan College of Traditional Chinese Medicine, Sichuan, China; 5 Department of Cardiac Surgery, Anhui Chest Hospital, Anhui, China

**Keywords:** clinical decision support, gold-standard case replay, heart failure, large language model, retrieval-augmented generation, risk-tiered autonomy

## Abstract

Heart failure (HF) care requires repeated decisions across suspected disease, diagnostic confirmation, phenotyping, guideline-directed medical therapy, device consideration, worsening HF, transition care, and advanced HF planning. Large language models (LLMs) may support this work by synthesizing structured and unstructured electronic health record data, retrieving current evidence, and presenting patient-specific reasoning. However, an HF-specific LLM clinical decision support system should not be framed as a single autonomous agent. We present the Heart Failure Intelligent Agent (HF-IA) as a modular, risk-tiered conceptual framework in which agent functions have different data requirements, reference standards, risk levels, and validation pathways. We argue that evaluation should combine node-level tests, longitudinal case replay, silent prospective validation, and post-deployment monitoring. This framework is conceptual and does not claim clinical effectiveness; its value is to clarify design, evaluation, and governance requirements for future LLM-enabled HF decision support.

## Introduction

Heart failure (HF) remains a major cause of morbidity, hospitalization, and health system burden worldwide (1, 2). Contemporary HF care is not a single decision but a longitudinal sequence of decisions made under uncertainty. Clinicians must integrate symptoms, physical findings, natriuretic peptides, kidney function, potassium, electrocardiography, echocardiography, ischemic evaluation, device data, comorbidities, patient preferences, and local resource constraints. The 2022 AHA/ACC/HFSA guideline and the 2023 ESC focused update illustrate how rapidly HF recommendations evolve, including pharmacological therapy, comorbidity management, device therapy, advanced HF referral, and care coordination (3, 4).

The complexity begins before HF is confirmed. Dyspnea and edema may reflect HF, chronic lung disease, kidney disease, anemia, obesity, venous disease, pulmonary hypertension, medication effects, or mixed syndromes. After HF is diagnosed, the clinician must identify phenotype and etiology, because HFrEF, HFmrEF, HFpEF, right-sided HF, valvular HF, infiltrative cardiomyopathy, myocarditis, tachycardia-mediated cardiomyopathy, and ischemic cardiomyopathy lead to different investigations and management pathways. The patient then moves through repeated cycles of treatment initiation, dose titration, laboratory surveillance, device consideration, worsening HF detection, discharge planning, transition care, rehabilitation, and, for some patients, advanced HF or palliative care planning.

Large language models (LLMs) have created interest in clinical decision support (CDS) because they can process free-text questions, summarize records (19), and combine natural language interaction with retrieval-augmented generation (RAG) (5–8). Yet medical LLM studies also show that benchmark performance does not by itself establish clinical safety (5–7). For HF, the risk is especially clear: a system that gives a reasonable educational answer may still be unsafe if it misses hyperkalemia, worsening kidney function, hypotension, device eligibility, or impending decompensation.

This Perspective is not intended as a systematic review of all LLM applications in cardiology or as a report of an implemented software product. Instead, we advance a design and evaluation framework: HF-specific LLM-CDS should be evaluated according to the clinical decisions it supports, the data it requires, and the harm that may result from error. We therefore present the Heart Failure Intelligent Agent (HF-IA) as a conceptual, modular, disease-specific, risk-tiered framework embedded in clinical governance.

## Why HF-IA should be modular rather than monolithic

The original temptation in LLM-based CDS is to describe an intelligent agent that assists with differential diagnosis, phenotyping, risk stratification, treatment adjustment, and follow-up. That description is directionally useful but technically insufficient. These tasks require different inputs, different reference standards, and different tolerances for error. Diagnosis depends on symptoms, examination, natriuretic peptides, imaging, and exclusion of mimics. Phenotyping requires ejection fraction, structural findings, ischemic and valvular assessment, rhythm, infiltrative disease clues, and comorbidities. Treatment optimization depends on blood pressure, heart rate, kidney function, potassium, volume status/congestion, drug history, adherence, frailty, pregnancy potential, cost, and patient preference.

HF-IA should therefore be conceptualized as coordinated modules: diagnostic support, phenotype and risk assessment, guideline-directed medical therapy (GDMT) safety and optimization, device eligibility screening, worsening HF detection, transition-care support, and patient-facing education. A shared orchestration layer can route queries, assemble the relevant patient context, retrieve evidence, apply safety rules, and return traceable recommendations. Data exchange should be designed around health-system standards such as FHIR when EHR integration is pursued (9). The clinical output should distinguish what is known, what is missing, what is inferred, and what action requires physician judgment.

The orchestration layer should not simply concatenate all EHR data into an LLM prompt. It should first define the decision context, identify required data elements, check whether those elements are present and current, and block or downgrade recommendations when critical information is missing. For example, a GDMT titration module should not recommend mineralocorticoid receptor antagonist escalation without recent potassium and kidney function. A device eligibility module should not infer CRT candidacy without QRS duration, morphology, ejection fraction, symptoms despite therapy, rhythm, and expected survival. A worsening HF module should treat rising creatinine differently depending on volume status, signs of congestion such as edema, weight gain, pulmonary congestion, or elevated jugular venous pressure, diuretic exposure, blood pressure, urine output, and recent medication changes.

This modular design is also important for evaluation. A drug titration recommendation cannot be validated by the same benchmark as a discharge follow-up reminder. A device eligibility module should be judged by guideline criteria and expert review, whereas a decompensation module should be judged for missed degradation, false alarms and subgroup performance. Treating HF-IA as a coordinated framework rather than as a single agent makes its claims more precise and testable.

## Mapping HF-IA to the heart failure course

A good HF-IA framework must follow the patient rather than a static checklist. When HF is suspected, it may help assemble evidence for or against HF and prompt evaluation for mimics. After diagnosis, HF-IA should support classification of HFrEF, HFmrEF, HFpEF, right-sided HF, valvular disease, ischemic cardiomyopathy, amyloidosis, myocarditis, arrhythmia-mediated cardiomyopathy, and other phenotypes when the available data suggest them. During treatment, it should identify opportunities and contraindications for GDMT, monitor kidney function and potassium, and explain why a recommendation is deferred when data are missing.

**Figure 1:**
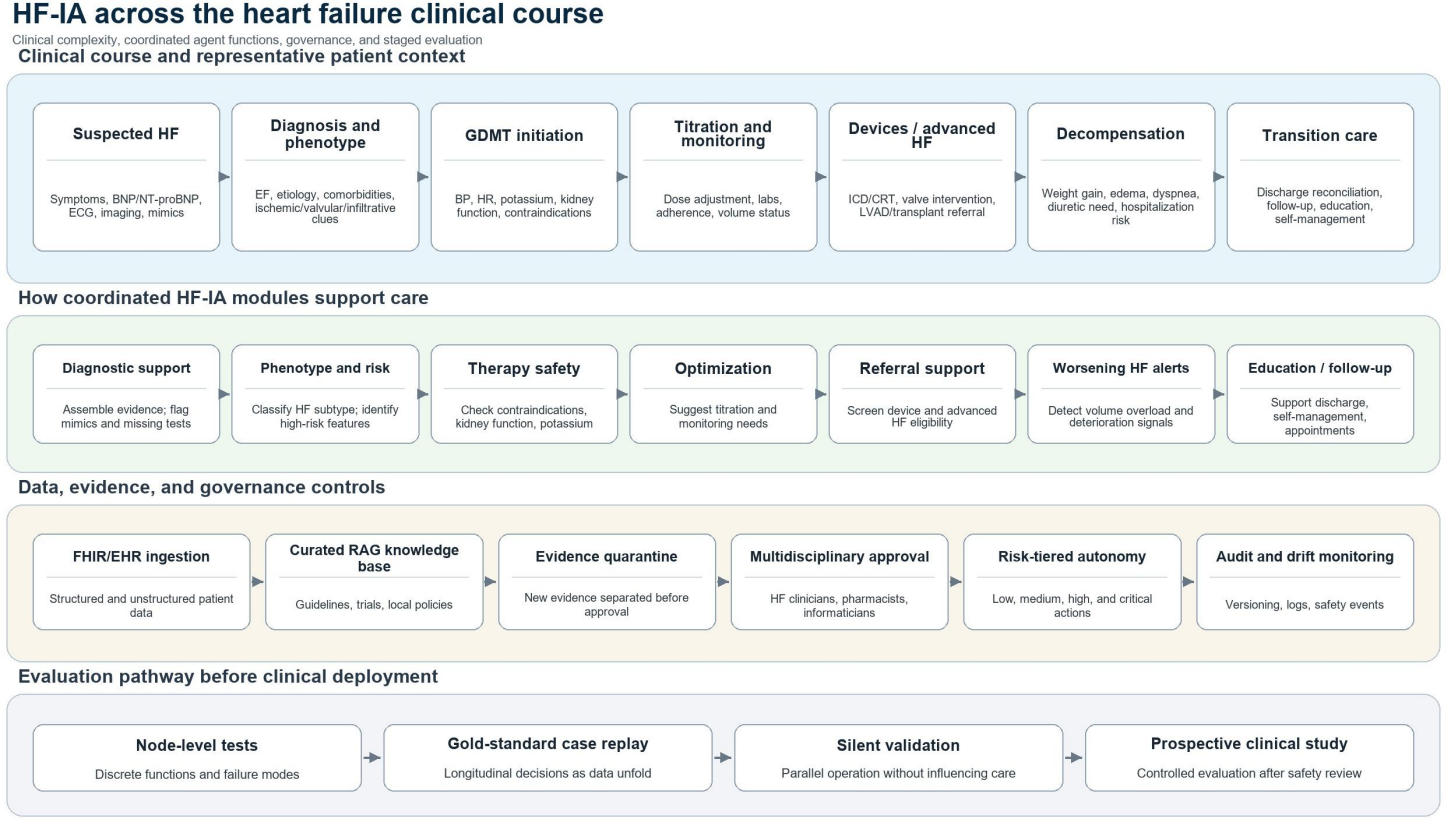
Proposed HF-IA framework across the longitudinal HF clinical course. The figure links major HF care stages with representative patient data, HF-IA functions, governance requirements, and staged evaluation. HF-IA is conceived as coordinated modules rather than a single autonomous agent.

Longitudinal management requires additional functions: detecting worsening HF from symptoms, weight, diuretic use, natriuretic peptides, kidney function trajectory, device alerts, and recent utilization; identifying eligibility for ICD, CRT, valve intervention, revascularization assessment, rehabilitation, iron therapy, sleep apnea evaluation, and advanced HF referral; supporting lifestyle counseling, sodium and fluid guidance when appropriate, vaccination reminders, exercise or cardiac rehabilitation planning, discharge reconciliation, early follow-up, and self-management education consistent with clinician-approved plans. Figure 1 summarizes this clinical and technical architecture.

## Evidence updating and guideline-conflict arbitration

An updatable knowledge base is useful only if its update process is governed. HF evidence changes faster than guideline cycles. Recent trials in HFpEF and HFmrEF, including SGLT2 inhibitor trials, obesity-related HFpEF trials, and newer mineralocorticoid receptor antagonist evidence, show why a simple guideline lookup system is inadequate (10–12). Conversely, automatically incorporating every new trial into recommendations would be unsafe.

We propose a staged evidence pipeline. First, evidence surveillance identifies new guidelines, randomized trials, meta-analyses, regulatory safety notices, and major society statements. Second, evidence triage classifies relevance by HF phenotype, population, intervention, comparator, outcome, and safety signal. Third, a critical appraisal layer grades certainty, directness, and applicability. Fourth, new material enters an evidence-quarantine state when it is promising but not yet incorporated into active clinical recommendations. Fifth, the governance group (HF clinicians, pharmacists, informaticians, methodologists) approves promotion, restriction, or rejection. Finally, versioning should record the source, date, rationale, approved use, and rollback pathway, consistent with broader principles of responsible machine learning deployment and clinical translation (13, 14).

The evidence pipeline should also handle conflicts explicitly. A new trial may show benefit in a population different from local patients, use a composite endpoint, exclude advanced kidney disease, or report safety concerns clinically important for older adults. In such cases, HF-IA should not collapse the evidence into a binary recommendation. Instead, it should present the active guideline recommendation, emerging evidence, the population to which the evidence applies, uncertainty, and why a clinician or governance group may choose to wait for guideline inclusion. When local formularies, reimbursement, or monitoring capacity limit implementation, the system should make these constraints visible rather than silently assuming ideal conditions.

HF-IA responses should expose this status.For example, a recommendation based on an active guideline should be labeled differently from emerging evidence that may inform specialist discussion but should not drive automated prescribing. This addresses the gap between vetted guidelines and new trial publications.

## Risk-tiered autonomy and human oversight

A blanket definition that all HF-IA output must be human-in-the-loop is too vague. HF-IA should instead use risk-tiered autonomy. Low-risk tasks such as flagging a missing follow-up appointment or creating patient education from an approved template can be automated with audit logs and clinician override. Medium-risk tasks such as suggesting overdue kidney function monitoring after medication adjustment should require clinician confirmation. High-risk functions such as diagnosis, medication initiation/titration, device eligibility, and worsening HF should remain human-in-the-loop. Critical events such as shock, severe hyperkalemia, acute pulmonary edema, or advanced HF decisions should be human-in-command with explicit emergency routes.

Risk level should depend on both the clinical action and deployment. Scheduling a follow-up visit is usually low risk, but it becomes higher risk when the patient has recent hypotension, rising creatinine, severe symptoms, or missed diuretic escalation. Patient education may be low risk when using a discharge template, but higher risk when taking symptoms or suggesting medication changes. HF-IA should assign risk dynamically depending on patient state, missing information, and the consequence of an incorrect recommendation.

This risk-tiered approach increases usefulness without weakening safety. It also provides a practical implementation path for health systems: begin with low-risk workflow support, then expand only after module-specific validation, usability testing, monitoring, and governance review. Table 1 summarizes the main HF-IA decision domains, autonomy levels, and evaluation focus.

**Table 1:** HF-IA decision domains, autonomy levels, and evaluation focus.

HF decision domain	Examples of HF-IA support	Minimum data context	Risk/autonomy level	Evaluation focus
Suspected HF and diagnosis	Prompt diagnostic work-up; flag mimics and missing tests	Symptoms, examination, ECG, natriuretic peptides, imaging, kidney function	Medium-high; clinician confirmation	Expert concordance; missed HF and mimic rate
Phenotyping and risk	Classify HFrEF/HFmrEF/HFpEF; identify ischemic, valvular, infiltrative, arrhythmia-mediated clues	Ejection fraction, echo structure, rhythm, ischemia/valve data, comorbidities	High; human-in-loop	Phenotype agreement; missing-data detection
GDMT safety and titration	Identify GDMT opportunities, contraindications, monitoring needs, and dose-safety issues	Medication history, BP, HR, eGFR, potassium, congestion, adherence, preferences	High; human-in-loop	Guideline concordance; potential harm and omission rate
Devices, worsening HF, transition care	Screen ICD/CRT or advanced HF referral; detect decompensation; support discharge and follow-up	EF, QRS, NYHA class, hospitalizations, weight, labs, device alerts, discharge plan	Low to critical; risk-tiered	Referral sensitivity; alert burden; readmission-related process measures

## Gold-standard case replay as the longitudinal core of HF-IA evaluation

We retain gold-standard case replay as the distinctive longitudinal evaluation strategy for HF-IA. Its purpose is to test whether the system updates reasoning appropriately as new information becomes available, which is central to HF care. However, gold-standard case replay should be anchored by parallel node-level testing rather than used as a stand-alone benchmark. Many individual decisions can and should first be evaluated separately before the full temporal sequence is replayed.

We propose a four-stage evaluation pathway. First, node-level evaluation should test discrete functions with expert-adjudicated cases: diagnostic classification, phenotype recognition, contraindication detection, GDMT opportunity identification, dose-safety checks, device eligibility, decompensation warning, discharge planning, and education quality. Metrics should include expert concordance, omission rate, potential harm rate, calibration, evidence traceability, missing-data detection, and subgroup performance. Second, gold-standard longitudinal case replay should feed de-identified data in the order originally available and compare HF-IA recommendations with expert-defined best actions at prespecified decision nodes. Third, silent prospective validation should run HF-IA in parallel with real care without influencing clinicians, allowing assessment of alert burden, false positives, workflow fit, and safety signals. Fourth, only after these stages should interventional studies be considered, following DECIDE-AI for early clinical evaluation and CONSORT-AI or SPIRIT-AI when clinical trials are planned (15–17).

The ground truth should vary by module. Diagnostic outputs should be compared with HF specialist adjudication supported by final clinical diagnosis, echocardiography, natriuretic peptides, and follow-up data. Phenotype outputs should be compared with structured expert labels and imaging results. GDMT outputs should be compared with guideline-concordant expert decisions considering contraindications, intolerance, kidney function, potassium, blood pressure, and patient preference. Device eligibility outputs should be compared with guideline and specialist review. Decompensation alerts should be compared using hospitalization, intravenous diuretic requirement, urgent visit, clinical adjudication, and time-to-event performance. Education and transition-care outputs should be checked for accuracy, readability, language appropriateness and consistency with the clinician-approved plan.

Evaluation should also include failure mode analysis. The most important errors may be omissions rather than incorrect statements: a missed potassium value, ignored worsening kidney function, missed QRS duration suggesting CRT referral, recurrent admissions that suggest advanced HF referral, or a general HFpEF answer that fails to recognize amyloidosis clues. Human factors are equally important. A model that produces correct but long recommendations may still fail if cognitive load increases, alert fatigue increases or if it fails to explain evidence provenance in a way clinicians can verify quickly.

No single public dataset is likely to cover the full HF course with complete decision inputs and outcomes. Future validation will require multicenter, de-identified*,* expert-adjudicated datasets with HFrEF, HFmrEF, HFpEF, acute decompensation, comorbid kidney disease, older adults, women, and underrepresented populations. Reporting should also reflect new AI-specific guidance for prediction and decision support models – transparent description of sources, intended use, human oversight, model updating (18).

## Discussion

This Perspective narrows the claim of HF-IA while strengthening its value. The proposed framework is not a proof that LLMs improve HF outcomes, but rather a design and evaluation argument: HF-specific LLM-CDS should be modular, evidence-governed, risk-tiered, and evaluated across both discrete decisions and longitudinal care. This is different from generic medical chatbots, single source RAG guideline tools and domain-agnostic agent frameworks because HF-IA is grounded in clinical course, data dependencies, and safety profile of HF management.

Several limitations should be explicit. First, HF-IA is conceptual. There is no deployed system, performance estimate, or patient outcome reported. The framework specifies what should be built and evaluated, not what has been proven. Second, the proposed modules require institutional data integration, FHIR-compatible interfaces, role-based access control, audit logs, cybersecurity testing, and local workflow adaptation. Third, knowledge updating requires governance resources that may not be available in all settings. Fourth, evaluation data sets must be diverse enough to avoid building a system that works only for a narrow HF population. Fifth, regulatory classification may vary depending on jurisdiction and by degree of autonomy and therapeutic influence.

These limitations are not minor implementation details. They define the boundary between a useful conceptual framework and a clinically deployable system. A hospital without reliable medication reconciliation, timely laboratory data, or structured echocardiography fields cannot deploy high-risk HF-IA modules without additional safeguards. Similarly, a system validated only in academic HFrEF patients should not be assumed to generalize to HFpEF, rural settings, older frail patients, advanced kidney disease, or patients with multiple comorbidities. Future work should therefore proceed in stages: prototype development, retrospective node testing, longitudinal case replay, silent prospective validation, usability assessment, governance review, and only then controlled clinical evaluation.

In sum, this Perspective offers a conceptual framework for developing and evaluating LLM-enabled CDS for adults with, or at risk for, HF. By mapping HF-IA to the complex clinical course of HF, the framework highlights how coordinated AI modules could support diagnosis, phenotyping, GDMT optimization, lifestyle and self-management support, monitoring, device referral, worsening HF detection, transition care, and advanced HF planning. Its central contribution is to connect HF-specific clinical decision points with evidence governance, risk-tiered autonomy, and staged evaluation. If developed and validated responsibly, such a framework may help physicians and health systems deliver safer, more consistent, and more scalable HF care while preserving appropriate human oversight.
